# Exercise Combined with Electrotherapy Enhances Motor Function in an Adolescent with Spinal Muscular Atrophy Type III

**DOI:** 10.1155/2019/4839793

**Published:** 2019-07-22

**Authors:** Massimiliano Gobbo, Sara Lazzarini, Laura Vacchi, Paolo Gaffurini, Luciano Bissolotti, Alessandro Padovani, Massimiliano Filosto

**Affiliations:** ^1^Department of Clinical and Experimental Sciences, University of Brescia, Brescia, Italy; ^2^Laboratory of Clinical Integrative Physiology, University of Brescia, Brescia, Italy; ^3^Department of Medicine and Surgery, University of Milano-Bicocca, Milan, Italy; ^4^Laboratory of Neuromuscular Rehabilitation, Teresa Camplani Foundation, Brescia, Italy; ^5^Functional Rehabilitation Service, Teresa Camplani Foundation, Brescia, Italy; ^6^Center for Neuromuscular Diseases and Neuropathies, Unit of Neurology, ASST “Spedali Civili”, University of Brescia, Brescia, Italy

## Abstract

**Background:**

Electrotherapy is widely used in physical therapy to increase muscle mass, improve motor function, and assist physical activity in several neurologic conditions. However, concerning Spinal Muscular Atrophy (SMA), limited evidence exists on the role of electrotherapy as an adjunct for improving muscle strength and function.

**Case Report:**

An adolescent (13 y.o.) with SMA type III underwent an 18-week strengthening program divided into two stages. During Phase I (weeks: 1-8), a home-based program for quadriceps strengthening through neuromuscular electrical stimulation (NMES) was provided. In Phase II (weeks: 9-18), at-home NMES was combined with functional electrical stimulation (FES) assisting volitional cycling for a broader, systemic conditioning. The treatment improved patient's structural and functional motor outcomes (quadriceps circumference and strength, Tinetti scale, and Hammersmith scale) as well as independence in stair climbing.

**Clinical Rehabilitation Impact:**

The purpose of this report is to raise awareness of the potential role of electrotherapy to help improving motor performance in SMA patients and, secondly, to foster further research aimed at assessing the actual contribution this intervention may have as an add-on therapy to existing care.

## 1. Introduction

Spinal Muscular Atrophy (SMA) is a chronic progressive neuromuscular disorder. Disease-related degeneration of spinal motor neurons results in reduced motor functions, muscle weakness, and atrophy. Despite its progressive nature, most children with type III SMA can stand and walk independently [1]. Nevertheless, the combination of proximal weakness and impaired balance results in frequent falls, leading to injuries and fractures [2, 3]. The consequent immobilization further worsens global functioning and autonomy. 

Electrical stimulation (ES) includes different techniques aimed at promoting motor recovery, limiting secondary symptoms (e.g., falls), and improving patients' quality of life (QoL). Extensive research testifies that ES is widely used in physical therapy to increase muscle mass, improve motor function, and assist physical activity in several neurologic conditions, such as stroke, spinal cord injury, and myotonic dystrophy [4]. Conversely, electrotherapy has not been thoroughly explored for SMA: to our knowledge, only one study tested ES effectiveness in SMA children [5].

The clinically relevant peculiarity of ES is that, when applied with proper intensities, this treatment modality is able to recruit faster motor units than isolated voluntary contraction and to impose a greater metabolic demand to the activated muscle fibers [6].

ES may be applied in isolation, combined with exercise or even superimposed to concomitant voluntary contractions in order to engage more muscle mass in the context of rehabilitation or sports training [6, 7]. It has been demonstrated that ES, specifically neuromuscular electrical stimulation (NMES) with or without voluntary exercise, can be in some cases more effective in preserving muscle function than voluntary training and conventional rehabilitation procedures [7]. Moreover, NMES may provide better results than volitional training for partially or totally immobilized subjects unable to perform volitional exercise or sustain an adequate volitional training intensity and duration. It seems that more impaired patients would even respond more effectively to NMES than less compromised ones [7]. It is noteworthy to mention that NMES, as an external modality of muscle activation, is inherently not affected by central fatigue and subject's motivation.

In this framework, the aim of the present case report is to describe the effects of a multimodal intervention for motor impairment in SMA, combining neuromuscular electrical stimulation and volitional exercise.

## 2. Case Report

### 2.1. History and Findings on Admission

E.B., a 13-year-old Caucasian boy with SMA type III, came to our attention in September 2014. Motor impairment had been evident since the age of 14 months. SMA was diagnosed at the age of 32 months through genetic tests. To slow down motor function progressive loss, after diagnosis E.B. periodically underwent physiotherapy cycles, including hydrokinesis and swimming. His medical history was characterized by a mild restrictive ventilatory defect, frequent falls, low physical endurance, and increased fatigability.

A fall occurred in May 2013 resulted in a fracture of the left femur metaphysis. The traumatic injury was fixed with a conservative treatment through a 6-week application of a plaster cast. In the following 3 months, the patient used an orthopedic support and attended an intensive rehabilitation program based on daily sessions of physiotherapy. Despite the treatment, recovery was incomplete: E.B. was able to walk independently but manifested lower limb weakness, decreased physical endurance, reduced gait speed, and increased fatigue and inability to climb stairs. This led to a reduction in autonomy and self-sufficiency with significant implications for his QoL.

In September 2014 at the first visit in our center (T0), physical examination showed remarkable atrophy of the thigh muscles, moderate scoliosis, absence of the patellar reflex with reduction of the other deep tendon reflexes, predominantly proximal muscle weakness (with the pelvic girdle more affected than the shoulder girdle), and inability to rise up from the floor. E.B. could stand and walk without support only for short paths and had a waddling gait with a compensatory exaggerated lumbar lordosis. Functional evaluation included quadriceps strength measurement through maximal voluntary isometric contraction (MVIC) [8], gait and balance evaluation through Tinetti scale [9], and motor functions evaluation through Hammersmith Functional Motor Scale-Expanded for SMA (HFMSE) [10]. During this initial assessment, E.B. underwent also transcutaneous neuromuscular electrical stimulation (NMES) to assess the presence of elicitable contractions of the quadriceps muscles and ascertain patient's tolerability to ES. Given the favorable response and good tolerance of the subject, we proposed him a personalized program for motor recovery centered on ES applications for lower limb strengthening. E.B.'s parents gave an informed consent for his participation to the program. At the end of the program, they signed a written informed consent for the publication of the results. The local Ethical Committee was informed about the case report.

### 2.2. Intervention and Management

The program was divided into two phases (Phase I; Phase II). Due to quadriceps weakness and deconditioning, EB was initially not able to properly exercise on the FES cycle-ergometer (i.e., maintaining cadence and power in the minimum range provided by the device) for more than 5 minutes. We therefore started with a preliminary (home-based) strengthening program to improve quadriceps force and power (Phase I).

Following recent ES recommendations, we defined the optimal electrode configuration for E.B.'s right and left quadriceps muscles (see later in the text for details), as well as the proper stimulus amplitude and parameters able to attain strong visible muscle contractions without discomfort [11, 12]. After a brief training to familiarize with the device and the technique, E.B. started a home-based NMES treatment protocol (Phase I; October-December 2014, 8 weeks). The adopted portable stimulator (Genesy 600, Globus, Italy) was used five times a week. Electrodes (size: 5x5 cm) were placed on the individualized motor points which were preliminary identified by mapping the muscle surface with a pen-electrode [12]. The motor points were marked on the skin and photographed.

The NMES protocol lasted 22 minutes and consisted of a warm-up phase (2 minutes), composed of single twitches (frequency = 5 Hz; stimulus width = 380 *μ*s). After the warm-up, a strengthening protocol (20 minutes) was provided with tetanic contractions (duration = 2 seconds; frequency = 35 Hz; and pulse width = 380 *μ*s) alternated by periods of recovery (9 seconds of single twitches at 3 Hz). The amplitude of stimulation was based on personal tolerance. Since the beginning, the stimulus amplitude was able to elicit well-tolerated strong visible contractions. E.B. was instructed to progressively increase the amplitude of stimulation each week in order to increase the muscular workloads. We supervised the treatment course through home telemonitoring and dairy compilation to continuously-constantly assess program adherence and NMES parameters as well as to check for potential side effects.

**Figure 1:**
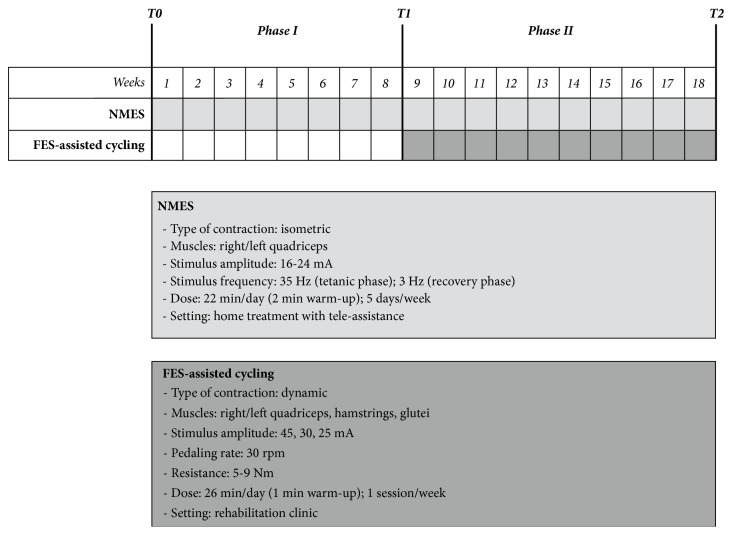
*Timeline of the program and description of the interventions. *NMES=neuromuscular electrical stimulation; FES=functional electrical stimulation; T0=baseline (beginning of Phase I); T1=after 8 weeks of treatment (end of Phase I; beginning of Phase II); and T2=end of treatment (end of Phase II).

After this initial muscle strengthening phase, we decided to add 10 sessions of voluntary cycling exercise assisted by functional electrical stimulation (FES-assisted cycling) with the aim of promoting broader beneficial effects (Phase II; December 2014-March 2015; 10 weeks). Once a week, E.B. went to our clinic to attend the FES-assisted cycling program. The FES cycle-ergometer (Pegaso, Biotech, Italy) provided coordinated bilateral stimulation to the quadriceps, hamstring, and gluteal muscles. The resulting evoked muscle activations support the patient's weak voluntary contractions during cycling. The protocol had duration of 25 minutes and was performed at the velocity of 30 rpm with pedal resistance gradually raised from 5 to 9 Nm. The stimulation amplitude was fixed at 45 mA for quadriceps, 30 mA for hamstring, and 25 mA for gluteal muscles. As for NMES, electrodes were placed on individualized motor points that were precisely searched with a pen-electrode. The device provided interactive videogame feedback in order to maintain motivation and increase volitional muscle engagement during the exercise. The ES program and the stimulation protocols used are resumed in Figure 1.

To evaluate the energy expenditure during FES-assisted cycling, oxygen consumption (VO_2_) and heart rate were recorded using a portable metabolimeter (K4b2, Cosmed, Italy). The attained exercise intensity was estimated through the calculation of the metabolic equivalents of task (METs). We adopted both standard and measured MET calculation. The standard MET value is classically computed by taking the energy costs (VO_2_ expressed in ml/kg/min) and dividing them by 3.5 ml/Kg/min, which is a proxy value for the resting metabolic rate of 1 MET [13]. Nevertheless, the level of physical activity estimated through standard METs may not be accurate for individuals with disability who may, actually, present resting VO_2_ values significantly lower than 3.5 ml/Kg/min [14]. For this, in order to provide more accurate estimates of the individual level of physical activity, we additionally computed the MET values by considering the measured VO_2_ during the resting state in sitting position (1 min). The VO_2_ peak value during FES-assisted cycling was the maximum VO_2_ value obtained by averaging each minute during the exercise session. The same procedure was adopted to calculate the basal and peak heart rate values.

### 2.3. Results

At the end of Phase I, after 8 weeks of home-based treatment (T1), quadriceps isometric strength significantly increased from 1.7 to 2.2 kg for the right side and from 0.8 to 2.0 kg for the left leg. The growth of thigh circumference (at 10 cm and 15 cm from the patella) was, respectively, 7 and 3 mm for the right leg and 5 and 3 mm for the left one.

At the end of Phase II (T2), thigh circumference (at 10 cm and 15 cm from the patella) grew compared to baseline, respectively, of 15 and 9 mm for the right leg and 12 and 6 mm for the left one. Dynamometry reported a significant gain in quadriceps isometric strength of both legs: MVIC increased by 70.6% for the right quadriceps and was nearly tripled for the left quadriceps (from 0.8 to 2.3 kg). Motor function evaluation by HFMSE showed an improvement of 7 points (from 35 to 42/66). Tinetti score increased by 8 points (+4 for balance, +4 for gait). Energy expenditure during FES-assisted cycling increased progressively from an initial value of 2.3 METs to a final value of 3.1 standard METs and from an initial value of 2.6 to a final value of 3.4 measured METs. Powers during cycling changed from 7 watts (average) and 14.4 watts (maximum) during the first session to 9.8 watts (average) and 16.8 watts (maximum) obtained during the last session. The main outcome measures are detailed in Table 1.

From the patient's perspective, E.B. always showed good treatment compliance and did not complain of pain, discomfort, or any other side effects during the whole treatment period. E.B. reported the maximum level of satisfaction (+2) related to the achievement of individual expected goals and to perceived treatment effectiveness (Goal Attainment Scale [15]). He never perceived excessive muscle fatigue and soreness. The patient reported improved gait abilities in terms of quantity, quality, and safety, as well as a reduced fear of falling. After the treatment, in March 2015, the subject recovered the ability to climbing stairs in complete autonomy. In light of these beneficial effects, E.B. applied for a 70% cofunding to acquire a FES cycle-ergometer. In June 2015, he obtained the device, which allowed him to continue the whole rehabilitation protocol at home.

## 3. Discussion

In this case report, we described the application of a treatment program, based on the combination of electrotherapy and cycling exercise, for the management of motor disability in a young patient with SMA type III. The novelty concerns different aspects. Firstly, we integrated muscle strengthening in static conditions (NMES) with a broader conditioning stimulation aimed at supporting and mimicking finalized movements superimposed to volitional pedaling (FES-assisted cycling). Secondly, we adopted a patient-tailored approach, featured by optimal electrode placement (i.e., over the individual muscle motor points) and stimulation amplitude (i.e., able to induce effective muscle contractions), avoiding treatment-related discomfort or side effects [11, 12]. Finally, the protocol has been purposefully designed in the perspective of conducting the treatment mostly at home, thus increasing the adherence to the treatment itself. In this light, indeed, ES devices may be affordable solutions to perform regular training in a comfortable and safe home environment.

Concerning the structural outcomes, NMES provided a substantial increment in thigh circumference (up to 7 mm at the end of Phase I) denoting muscular mass gain. This finding likely represents the underlying structural adaptation responsible for the enhancement in quadriceps strength, as revealed by the change in MVIC at the end of NMES home-based treatment (in particular for the left side). The aforementioned outcomes are in contrast with previously published data [5]. The conflicting results are probably due to the fact that in the present report, the adopted intensity of stimulation was beyond the motor threshold. Previous data, on the contrary, were obtained with stimulation intensities below the motor-threshold, namely, without eliciting muscle contractions.

From the functional point of view, the HFMSE scale showed an improvement especially in the items addressing standing performance and lower limb motor competences. Similarly, the Tinetti scale allowed appreciating a substantial gain in balance and gait. Both findings are in line with the achievement of higher strength levels developed by the knee extensors.

Stair management is the major obstacle for independent mobility for SMA type III patients [1]; thus, the recovery of this function is of paramount importance with relevant implications in daily living activities. After the 18-week treatment, E.B. recovered the ability and self-confidence to climb stairs, showing an overexpected treatment satisfaction and regaining possession of his personal spaces. The home-based nature of NMES and the activity-promoting videogame during FES-assisted cycling likely contributed to patient's compliance, full adherence to the proposed treatment, and constant motivation in progressively increasing the workload.

A final consideration pertains to the fact that, besides local effects, muscle contractions evoked by NMES and the adapted volitional physical activity assisted by FES may provide systemic beneficial effects. Indeed, skeletal muscle is now fully considered as a secretory organ that produces circulatory factors (myokines) during contraction. Myokines consist of several secreted peptides which are thought to mediate multiple beneficial effects (anti-inflammatory, lipolytic, anabolic, osteogenic, cardioprotective, and neurotrophic) and protect against chronic diseases [16]. FES-supported leg exercise is able to increase whole-body metabolism [17] and improve glucose and lipid metabolism [17, 18], thus contributing to ameliorate the individual fitness status. In parallel, prolonged aerobic exercise, favored in this case by FES-assistance, may decrease the incidence of cardiovascular disease by reducing inflammatory markers such as IL-6, TNF-alpha, and CRP [18]. In particular, the metabolic expenditure achieved by E.B. during FES-cycling training exceeded 3 METs (for both measured and standard estimations), which represents the threshold level for classifying an exercise as “moderate physical activity” [19]. Moderate activity has been thoroughly associated with beneficial cardiovascular and metabolic effects and therefore highly recommended for health promotion [19].

## 4. Methodological Considerations

Following recent ES recommendations, we paid particular attention in defining the optimal electrode configuration for E.B.'s right and left quadriceps muscles, as well as the proper stimulus amplitude and parameters able to attain strong visible muscle contractions without discomfort [12]. This aspect is remarkably critical when applying NMES for strengthening purposes in that an effective mechanical stimulus is essential to induce new contractile protein synthesis and increase muscle fiber diameter [11].

## 5. Limitations

Some limitations of the study have to be discussed. First, as a case report, the external validity of the presented results and the level of evidence of the study are limited. Secondly, during Phase II, we used the combination of electrotherapy and exercise. We were not able to assess the relative contribution of the different modalities included in the second part of the program and therefore we do not know what specific benefit each intervention had as a part of the treatment. We presume that volitional activation of leg muscles during cycling was mainly responsible for the systemic conditioning and related increase in METs value, while ES mainly contributed to initial quadriceps strengthening.

## 6. Conclusions

Alternative and advanced models of intervention are constantly needed to enhance or maintain motor performance, self-dependence, and QoL of patients with neuromuscular diseases. The findings of this case report, although with limited generalizability, reveal that a multimodal approach based on electrotherapy and cycling exercise may be well tolerated and may improve motor performance in SMA patients, thus contributing to designing more robust experimental studies aimed at investigating the effectiveness of ES as an add-on therapy to existing and emerging care.

## Figures and Tables

**(a):** 

	Thigh circumference (cm)				MIVC (Kg)		Tinetti scale			HFMSE
	10 cm from patella		15 cm from patella							
	Right	Left	Right	Left	Right	Left	Gait	Balance	Total	
T0	38.9	39.0	40.3	40.5	1.7	0.8	7/16	8/12	15/28	35/66
T1	39.6	39.5	40.6	40.8	2.2	2.0	11/16	10/12	21/28	NE
T2	40.4	40.2	41.2	41.1	2.9	2.3	11/16	12/12	23/28	42/66

**(b):** 

	HR (bpm)		VO_2_ (ml/Kg/min)		Measured METs	Standard METs
	Basal	Peak	Basal	Peak		
T1	87	101	3.12	8.11	2.6	2.3
T2	86	112	3.03	10.9	3.4	3.1

(a) T0=baseline (beginning of Phase I); T1=after 8 weeks of treatment (end of Phase I; beginning of Phase II); T2=end of treatment (end of Phase II); MVIC=maximal voluntary isometric contraction; HFMSE=Hammersmith Functional Motor Scale-Expanded; NE: not evaluated; (b) Values calculated during the first and the last session of the FES-assisted cycling exercise program; HR=heart rate; VO_2_=oxygen consumption; and METs=metabolic equivalents of task (measured METs were calculated as the ratio of the peak metabolic rate to the measured basal metabolic rate in quite sitting position; standard METs were calculated as the ratio of the peak metabolic rate to a standard resting metabolic rate equal to 3.5 ml/Kg/min).
